# Many Interesting Paragraphs

**Published:** 1894-05

**Authors:** 


### Medical News and Miscellany.

**Dr. A. Jay Sibley** has located at West Point, Texas.

**Dr. J. W. Carhart** has removed from Lampasas to La Grange, Texas.

**Dr. W. B. Anderson**, who has just completed a course at the New Orleans Polyclinic, has located at Taylor, Texas.

**Dr. W. L. York**, of Decatur, Texas, although absent, was not forgotten; he was elected 1st vice-president; a rare compliment.

**Dr. J. D. Osborn** was elected mayor of Cleburne last month. What do so many doctors want to be mayor for? Dr. Fly, of Galveston, has made and is making a good mayor.

**Dr. R. B. McKinney**, of Memphis, Tenn.,—a protege of Prof. Sim, and connected with him on the *Memphis Medical Monthly*, has gone to Germany to finish his medical education. We predict he will "make his mark" and be a professor, some day. He is a fine young fellow.

**Dr. P. C. Coleman**, of Colorado, will be the next President of the Texas State Medical Association. He will be elected at Dallas; mark the JOURNAL'S prediction. Dr. Coleman is a most popular and courteous physician and is a good speaker. He has claims for promotion which will not be ignored.

**The Journal** is pleased to announce that Dr. Seth M. Morris, Professor of Chemistry in the Texas Medical College, who for two years has had his appointment only for a year at a time, was, at the last meeting of the Regents, made full professor and put upon same footing as the other members of the faculty.

**A Hot Supper.**—While the disappointed ones who got badly left on the second round at that dismal failure of a banquet were consoling each other, dear, good old Dr. Sears, the late President, made them all feel worse by telling them he had "had a hot supper,"—he "got a cracker and some pepper sauce," he said.

**Dr. Robert Towns Morris,** of Austin, a grandson of the venerable Dr. W. A. Morris, graduated with distinction at the Tulane University Medical College last month; his thesis—Puerperal Eclampsia—was one of three selected by the Faculty out of one hundred, for publication. It will appear in the next number of the JOURNAL.

**Dr. T. J. Tyner** returned from Rome in time to take part in our big State meeting. He read a paper at the International Medical Congress, on his operation for cataract, which was much complimented. It was translated into German and Italian, and was published in some of the European journals. We congratulate the doctor on his success.

The American Medical Editors' Association will hold their annual meeting at the Palace Hotel, San Francisco, California, Monday evening, June 4th, prox., C. H. Hughes, M. D., President; Geo. M. Gould, M. D., Secretary; I. N. Love, M. D., Chairman Committee of Arrangements, and a banquet has been tendered them by Mr. R. E. Queen, of San Francisco. Of course they will have a good time; they always do.

